# Super-pulsed Diode Laser in the Therapy of Inferior Alveolar Nerve Paresthesia After Mandibular Third Molar Extraction: A Case Report

**DOI:** 10.7759/cureus.76147

**Published:** 2024-12-21

**Authors:** Aubert V Brito, Arlyn Angulo, Ricardo Almon

**Affiliations:** 1 Patologia Bucal y Cirugia Maxilofacial, Hospital Jose Gregorio Hernandez, Caracas, VEN; 2 Oral Medicine, IPD Institute, Caracas, VEN; 3 Oral Medicine, Almón Brito IPD (Implantology, Periodontology, Oral Diagnostic) Institute, Caracas, VEN; 4 Implantology and Periodontology, Almón Brito IPD (Implantology, Periodontology, Oral Diagnostic) Institute, Caracas, VEN

**Keywords:** injured inferior alveolar nerve, oral paresthesia, oral surgery, photobiomodulation, super-pulsed laser

## Abstract

Oral paresthesia occurs when one of the nerves in the region is injured, usually the inferior alveolar and/or lingual nerve, after dental procedures such as the extraction of lower third molars. The objective of this study was to describe the case of a patient who received photobiomodulation (PBM) therapy for paresthesia of the inferior alveolar nerve (IAN) caused by the extraction of mandibular third molars. The protocol used involved a super-pulsed diode laser with dual wavelengths of 810 nm and 980 nm, 1 W, 60 seconds, 12.15 J/cm², with a spot size of 25 mm in the extraoral area. In the intraoral region, 0.3 W, 60 seconds, 46.77 J/cm², with a 7 mm tip, was applied. Before starting each PBM session, mapping of the area was performed to delineate the regions with paresthesia, and the degree of sensitivity was evaluated using a visual analog scale (VAS). On the first day, before the first laser therapy session, the VAS assessment was 7, indicating partial loss of sensitivity. After 48 hours and before the second PBM session, the patient reported a slight tingling sensation and substantial improvement in chin sensitivity, with a VAS score of 4.5. After nine sessions, the patient reported recovery of sensitivity in all affected regions (VAS = 0), with positive and normal responses to touch with a dental explorer. Within the parameters established for this clinical case, our results suggest that PBM therapy may improve the loss of sensation observed in IAN paresthesia following injury or surgical trauma.

## Introduction

Oral paresthesia occurs when one of the nerves in the facial area is injured due to contact with or proximity to the region involved in dental procedures, such as the extraction of lower third molars, orthognathic surgery, and surgery for the placement of dental implants. The most commonly affected nerves are the inferior alveolar and/or lingual, with an incidence of 0.2 to 20% following the extraction of third molars and 40% after the placement of implants. The main symptoms include the absence or partial loss of sensitivity in the affected region, although tingling, itching, numbness, or burning sensations may also occur [1,2].

Currently, there is no standardized protocol for the evaluation and management of patients with paresthesia due to inferior alveolar nerve (IAN) injury, nor is there any surgical or pharmacological technique that guarantees complete healing of this damage. Treatments include drug protocols, such as vitamin B complex, and therapies like acupuncture, electrostimulation, physiotherapy, and humid heat. Surgical modalities are also employed for nerve repair, including epineural repair, interpositional nerve grafts, and vein grafts, with or without the application of neurotrophic factors on the IAN [3-5].

Recently, laser photobiomodulation (PBM) has become an increasingly utilized complementary treatment modality, particularly in the fields of physical medicine and rehabilitation. PBM is a non-thermal phototherapy aimed at modulating tissue metabolism. It uses red or near-infrared light (NIR) or light-emitting diodes (LEDs) to heal, restore, and stimulate multiple physiological processes and repair damage caused by injury or disease [1,6].

Red and NIR light wavelengths can influence the bioenergetic characteristics of mitochondria by interacting with photoreceptors such as cytochromes, water, lipids, S-nitrosylated nitric oxide (NO), and transient receptor potential channels (TRPC) for Ca²⁺ through various mechanisms. Stimulated cytochrome c oxidase (CCO) induces an increase in the activity of the electron transport chain and ATP synthesis, activating multiple signaling pathways that modulate cellular reactions [1,7]. The outcomes of the direct or indirect communication between light and mitochondria include the modulation of ATP and ROS production, the release of NO, and Ca²⁺ homeostasis [1,6-9].

The ability of light to penetrate tissue and deposit energy depends on the properties of the absorption and scattering centers that are randomly distributed. The scattering and absorption behavior depends on the wavelength of light [10,11], which governs its depth of penetration into tissue. The absorption and scattering coefficients are greater at shorter wavelengths; therefore, NIR penetrates deeper than red light [7-11]. This knowledge has allowed PBM therapies to evolve in their application for the treatment of various diseases and injuries, such as wound healing, musculoskeletal conditions, neurorehabilitation, and in the fields of oral medicine and veterinary medicine.

PBM has demonstrated several positive effects on the regeneration of peripheral nerve injuries. These include axon growth and myelination [1,7], reduction or prevention of scar formation, decreased mononuclear inflammatory infiltration, positive regulation of neurotrophic growth factors, improved functionality and enhanced neurosensory recovery [8], stimulation of adjacent or contralateral nervous tissues, and biomodulation of the nervous response at the normal threshold of the action potential [2,10].

There is a wide variety of PBM protocols for parameters such as wavelength, energy density, power density, and treatment frequency for therapeutic purposes and neurorehabilitation [1,5,6,11-14]. Light parameters and applied doses are fundamental in PBM therapy and refer to the use of light in the red or NIR region, with wavelengths usually in the range of 600 to 700 nm and 780 to 1100 nm, with an irradiance or power density between 5 mW/cm² and 5 W/cm². This type of irradiation can be a continuous wave or a pulsed light consisting of a relatively low-density beam (0.04 to 50 J/cm²), but the output power can vary widely from 1 mW to 500 mW to avoid thermal effects [6]. Most published studies on PBM are conducted with continuous-wave lasers, and few with dual super-pulsed lasers for the treatment of injuries in the IAN. The selection of the protocol to be applied will depend on the severity of the injury and the objective of the treatment; therefore, each case will be individualized. Additionally, it is important to understand the parameters of the equipment to be used, as they differ depending on the model and brand.

The objective of this study was to describe the case of a patient who received PBM therapy using a super-pulsed diode laser for paresthesia of the IAN caused by the extraction of mandibular third molars.

## Case presentation

**Figure 1:**
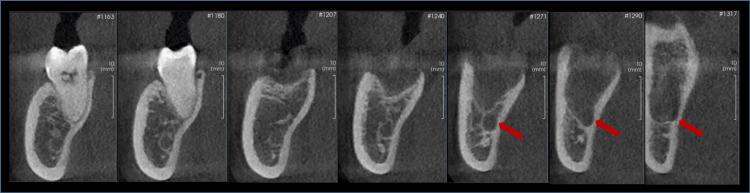
Cone beam tomography; sagittal section showing a hypodense image corresponding to the alveolus, with proximity to and communication with the inferior alveolar nerve (red arrows).

A 27-year-old female patient was referred to the dental consultation at the Almón Brito IPD Institute, Caracas, Venezuela, to the laser therapy department due to a lack of sensitivity in the anteroinferior left area. During the first consultation, after taking the medical history and conducting a physical examination, the patient reported having undergone surgery for the extraction of the lower third molars four weeks prior. Since then, she has experienced a lack of sensitivity in her lower lip (left side), extending to the chin, beneath it, and in the perioral regions on the left side. A cone beam tomographic study performed after the extraction of the third molar revealed communication between the alveolus and the IAN canal (Figure 1). The internal area of the mouth showed affected dental regions from the second premolar to the central incisor, as well as the lower gum on the buccal side. The patient noted some improvement in the paresthesia following a cycle of vitamin B injections previously prescribed by the surgeon who performed the extractions.

Once the evaluation of the clinical and radiographic data was completed, the patient's diagnosis was paresthesia of the IAN following the extraction of the lower left third molar. Consequently, low-intensity laser therapy was recommended three times a week with a 48-hour interval between sessions. Before each PBM session, mapping of the area was performed to delineate the regions with paresthesia. The mapping was carried out using a dental explorer, and the test consisted of gently pricking the face while asking the patient if she perceived the prick. Sites where the response was affirmative were marked in red.

To determine the degree of paresthesia, visual analog scale (VAS) tests were used as a subjective assessment, along with a deep sensitivity evaluation using the tip of a dental explorer [1]. The VAS is a widely accepted tool for standardizing or quantifying symptoms and complaints [5,15]. Patients marked points on the line that they felt represented their perception of their current state. The score was determined by measuring, in millimeters, the distance from the left end of the line to the marked point. A score of 0 indicated a complete absence of sensation, while 10 at the extreme end represented fully normal sensation.

**Figure 2:**
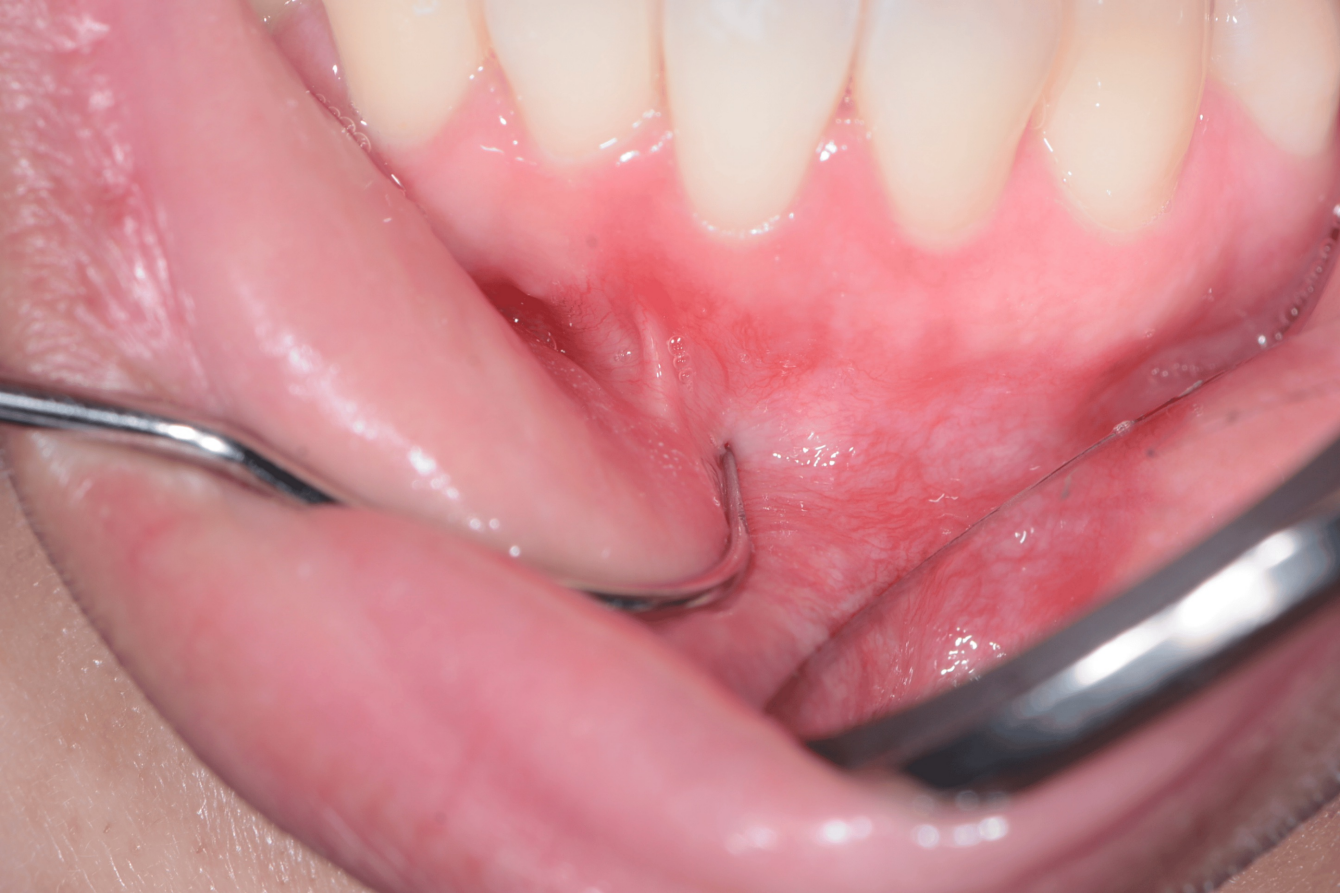
Examining the affected area using the tip of an dental explorer.

A deep sensitivity evaluation was conducted by a single operator to delineate the affected area. Using the tip of a dental explorer, the operator examined both affected and unaffected areas, asking the patient if she felt any kind of sensitivity (Figure 2). Subsequently, mapping of the area with paresthesia was performed to guide the treatment application. These tests were conducted at the beginning of each treatment session, just before irradiation. Fourteen days after completing the scheduled PBM sessions, the final evaluation was carried out.

**Figure 3:**
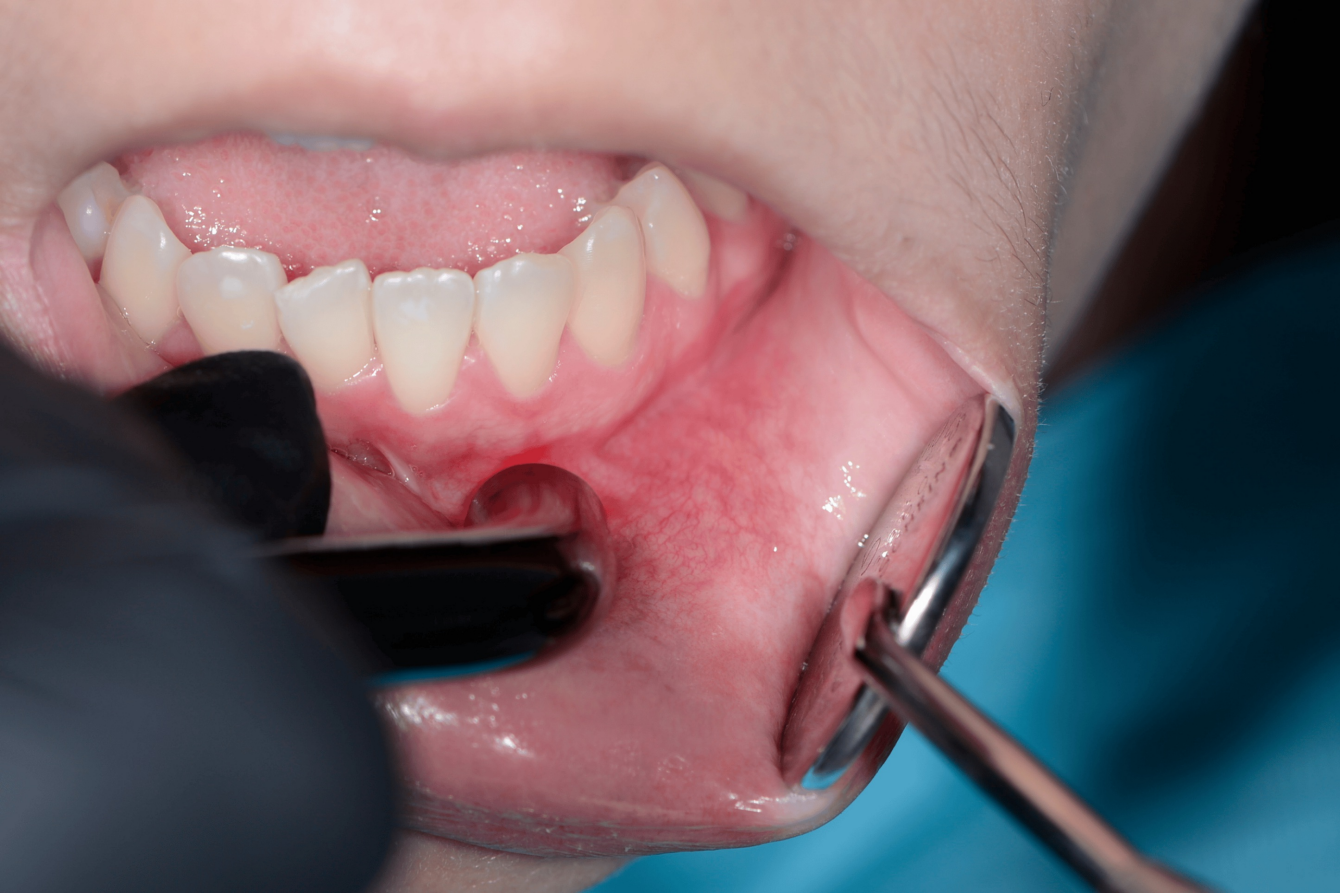
Photobiomodulation using a sweeping technique in the intraoral area.

**Figure 4:**
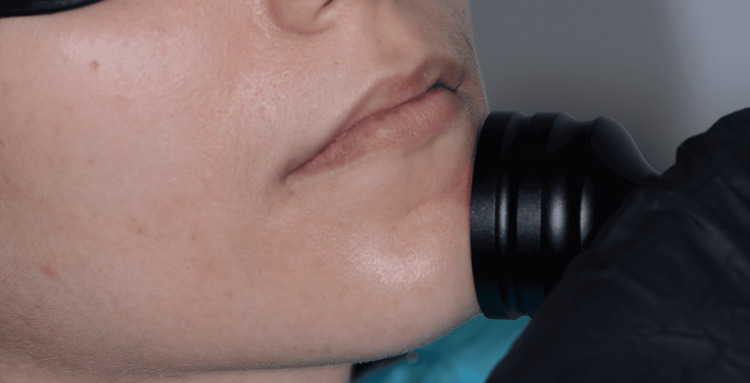
Photobiomodulation using a sweeping technique in the extraoral area.

The diode laser device used was the Gemini EVO™ (Ultradent Products, Inc., South Jordan, Utah) with dual wavelengths of 810/980 nm, a super-pulsed beam, a power of 1 W, a duration of 60 seconds, and a fluence of 12.15 J/cm² with a spot size of 25 mm in the extraoral area, applied using a sweeping technique. In the intraoral region, a power of 0.3 W was used with a 7 mm tip, a duration of 60 seconds, and a fluence of 46.77 J/cm², applied in a sweeping motion from the retromolar trigone region toward the vestibular sulcus to irradiate the IAN, the mental nerve path, and the labial mucosa. In the extraoral area, irradiation was performed in a sweeping motion in direct contact with the region, with the laser beam perpendicular to the tissue (Figures 3, 4). Intraorally, the path of the inferior dental nerve and chin region was followed, maintaining an approach distance of 1 cm. Before starting the laser treatment, the skin of the patient's face and oral mucosa was cleaned with sterile gauze to eliminate any interference of the laser beam caused by makeup, sweat, or saliva.

The patient signed an informed consent form, through which she authorized the use of the data generated during the study for academic purposes, including oral presentations and publications.

On the first day of treatment, before performing the first laser therapy session, the VAS sensitivity evaluation was 7, indicating a partial loss of sensitivity. After 48 hours, and before the second laser therapy session, the patient reported a slight tingling sensation and an improvement in chin sensitivity, with a VAS score of 4.5 (Table 1).

**Table 1:** Assessments of sensitivity during treatment using visual analog scale (VAS) test.

Assessments	VAS
Time 0 - first evaluation	7
Second evaluation	4.5
Third evaluation	3.6
Ninth evaluation	1.4
Final evaluation after 9 sessions	0

**Figure 5:**
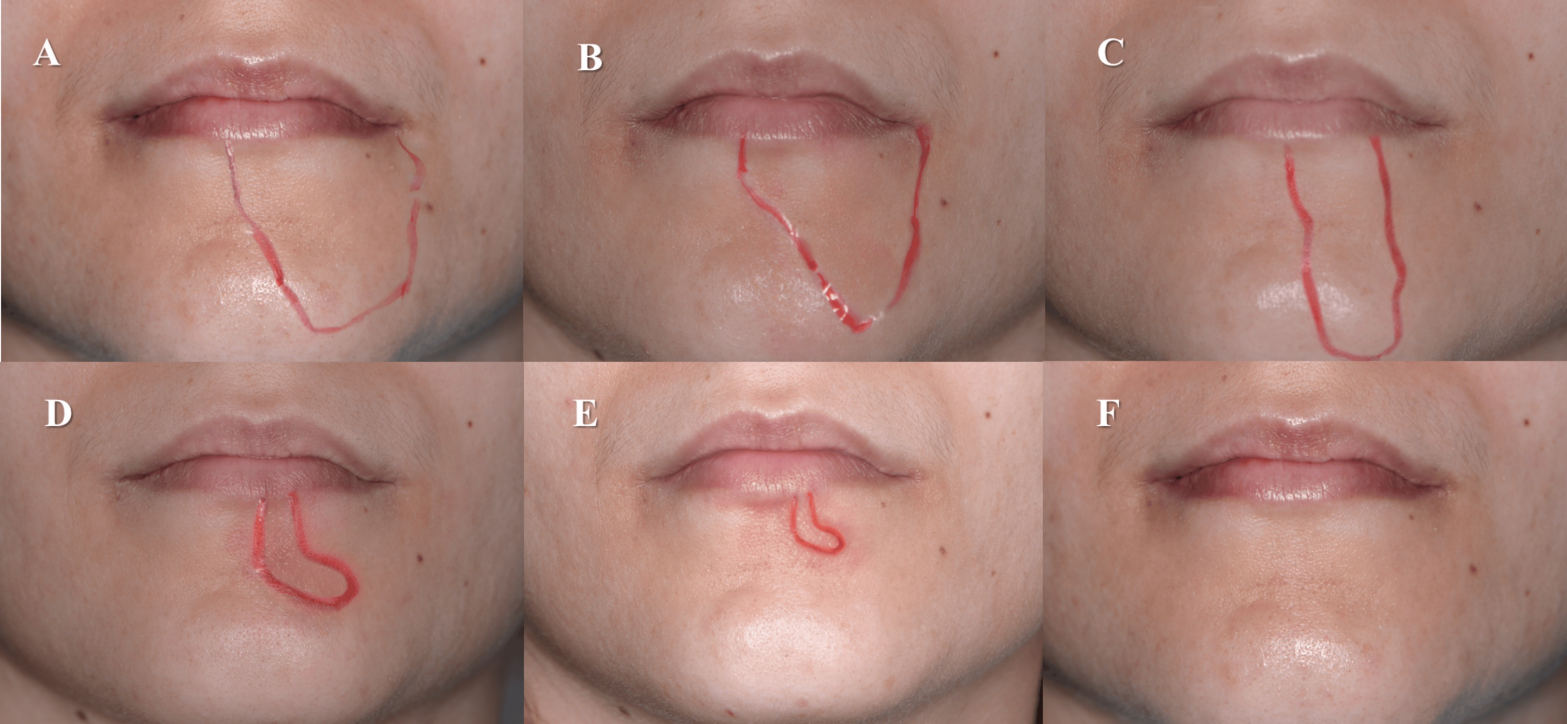
Evolution of the area with paresthesia during PBM treatment: (A) first appointment, (B) second appointment, (C) third appointment, (D) sixth appointment, (E) ninth appointment, (F) final evaluation (after 14 days).

After nine sessions, the patient reported a recovery of sensitivity in all areas previously affected by paresthesia. The VAS sensitivity test showed a value of 0, and she responded positively to touch with the dental explorer (Figure 5).

## Discussion

Dental procedures such as oral and maxillofacial surgeries, local anesthesia, extraction of third molars, implants, orthognathic surgery, and rigid internal fixation of fractures can cause damage to the trigeminal nerve. Among these, damage to the IAN is the most common complication [13]. This is generally due to the anatomical proximity between the roots of the lower third molars and the IAN.

Paresthesia is usually a temporary injury, but the resulting changes can alter functions such as speech, chewing, and social interactions. The time required for recovery can vary between several months and years. When paresthesia persists for more than six months, it is considered an intolerable morbidity for the patient. Therefore, early treatment is important for improving quality of life. The first therapeutic option for IAN injury is conservative treatment with supplements such as vitamin B and physical therapy. Unfortunately, the recovery period is often very long, which affects patients socially and psychologically [14,16,17].

PBM has been proposed as a useful complementary treatment modality for IAN paresthesia, especially in patients following dental surgery. However, the clinical therapeutic effects of PBM seem to depend on the precise treatment parameters of the device (wavelength, power density, energy density, and time), which are still unclear. It is not yet possible to provide comprehensive and informative instructions for the use of PBM in neurorehabilitation [12]. Additionally, the site of the injury after third molar surgery is located in the deep posterior part of the mandible, where the cortical bone may be too thick for light to easily penetrate [1,8,14].

This report presents the case of a 27-year-old female patient with paresthesia in the lower left anterior area for four weeks, as a consequence of the extraction of the lower third molar on that side. PBM therapy was indicated three times a week with an interval of 48 hours between sessions; nine sessions were performed. Despite the limited studies and divergences in the literature on irradiation and dosimetry protocols using super-pulsed diode lasers, it is important to highlight that the parameters used in this case were efficient. A protocol with an NIR wavelength laser was selected, where the light is invisible and falls within the range of 600 to 1000 nm, which allows greater penetration into biological tissues, essential for reaching and stimulating deep nerves. In PBM therapy, the minimum power used is typically between 0.1 and 0.2 W, except for large extraoral areas where larger tips are used, in which case the power is increased from 1 to 2 W. Depending on the diameter of the selected tip, the power density varies according to the area being treated; the larger the area, the lower the power density, and vice versa. For the treatment of this patient, doses of 1 W, 60 seconds, and 12.15 J/cm² were applied with a 25 mm spot size in the extraoral area. In the intraoral region, a power of 0.3 W and 60 seconds were used, with a 7 mm tip and a fluence of 46.77 J/cm².

Ravera et al. [8] conducted a review to address the question of which PBM therapy is most effective in the recovery of the branches of the trigeminal nerve. They evaluated various in vitro and clinical studies and concluded that PBM at 808 nm and 0.2 W/cm² (0.2 W; 12 J/cm²; 12 J per point; 60 seconds, continuous wavelength) proved to be a reliable therapy. Irradiations in both pulsed and continuous wave modes were found to affect IAN nerve regeneration and neurosensory recovery when applied with accurate wavelengths and doses.

Among the published cases using laser PBM therapy with similar protocols is that of Pol et al. [5], who applied a low-intensity super-pulsed GaAs diode laser using two wavelength sources: the first between 904 and 910 nm and the second at 650 nm, for neurosensory recovery of the IAN in 57 patients, obtaining good results in 83.3% of cases. The energy dose used was 123 J, with a frequency of 20 kHz, emitted over 900 seconds (15 minutes) per session. The power density was 0.27 W/cm², and the fluence was 244.8 J/cm². The cumulative dose for the entire treatment was 1230 J (123 J per session over 10 sessions performed weekly). They concluded that PBM therapy improves both subjective and objective deficits in neurosensory sensitivity observed in IAN paresthesia. The laser device used in their study differed from the one in our work. Although both generate laser light, the source of generation is different. We used a super-pulsed beam because it generates less heat in tissues [1], with different dosimetry. The dose used was higher but its application was more spaced than the one carried out in this case, which was every 48 hours with a cumulative dose of 540 J extraoral and 162 J intraoral.

Fernandez-Neto et al. [2] also reported a successful case treated with 26 sessions of PBM at 808 ± 10 nm, 100 mW, 3 J per point for 30 seconds per point, in continuous mode, twice a week. In this case, the laser device, light beam, and doses applied differed from those reported in our study. Although the ages of the patients were similar, their cases required more sessions than those applied in our report, possibly due to the longer time elapsed between the IAN injury and the initiation of PBM therapy.

Oliveira et al. [17] evaluated a therapeutic protocol using 808 nm or 660 nm, 100 mW, 100 J/cm², 2.8 J per point over 28 seconds per point in 125 patients with loss of sensitivity after orthognathic surgery. They observed that recovery correlated with age and the time interval between surgery and the initiation of therapy, concluding that PBM treatment positively affects sensitivity after oral surgery and that early treatment reduces the number of sessions required. These conclusions align with the positive results obtained in our patient, who was a young adult, and the time elapsed from the surgical trauma to the start of treatment was no more than four weeks.

The positive results reported across different studies suggest that lasers can stimulate sensory fibers of undamaged collateral nerves to grow towards areas of paresthesia or stimulate regeneration in injured nerves [5,7,15]. Thus far, the main advantage of PBM therapy for paresthesia is the absence of side effects such as pain. However, it must be administered by trained and certified professionals [2]. Potential side effects for patients include swelling, inflammation, redness of the skin, scar formation, changes in tissue pigmentation, and infection after treatment. These complications can be minimized by carefully following proper instructions during treatment.

## Conclusions

Within the parameters established for this clinical case, our results suggest that PBM therapy may improve the loss of sensitivity observed in IAN paresthesia following injury or surgical trauma. It is essential to establish criteria and protocols for the application of PBM therapies with the aim of recovering lost sensitivity caused by surgical trauma.
